# Initial in vivo validation of real-time phase-contrast sequence

**DOI:** 10.1186/1532-429X-17-S1-P393

**Published:** 2015-02-03

**Authors:** Bob S Hu, Michelle M Nystrom, Reeve Ingle, William R Overall, Michael Cates, Juan M Santos

**Affiliations:** 1 HeartVista, Inc., Menlo Park, CA, USA; 2 Cardiology, Palo Alto Medical Foundation, Palo Alto, CA, USA

## Background

Phase-Contrast MRI (PC-MRI) is a standard tool used for the quantitation of flow in everyday cardiac MR studies. However current methods require multiple breath-holds and post-acquisition analysis. A PC-MRI sequence that provides real-time acquisition and display of quantitative flow rates has the potential to substantially reduce scan times while providing the clinician with the immediate ability to query flow trends and disturbances. The feasibility of using real-time MRI for the quantitation of flow has been previously reported (Joseph, *et al*. JMRI 2014, 40:206-213). We have implemented a real-time sequence using the Heartvista cardiac package (HeartVista, Inc., Menlo Park, CA) for this initial, in vivo validation study.

## Methods

**Figure 1:**
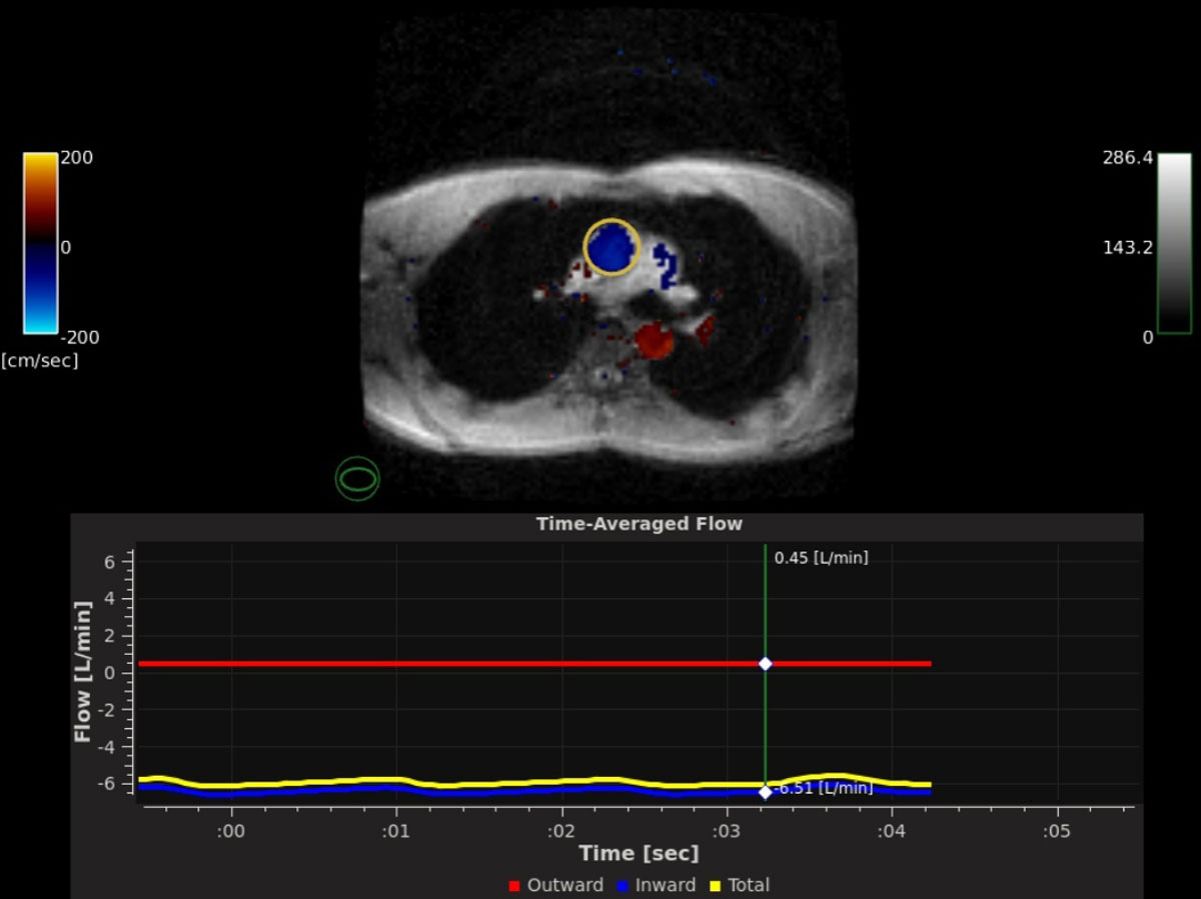
**A sample DICOM result from the real-time PC-MRI sequence.** The Phase-Contrast data is acquired in real-time and overlaid on GRE magnitude images (top). The time average flow of the phase-contrast data is measured within an ROI (yellow circle) and displayed on a graph in real time (bottom).

12 subjects (6 cardiac patients, average age 61; 6 volunteers, average age 36; 5 female, 7 male) were scanned in a GE 1.5T HDxt Twinspeed MRI. For each subject, the standard Fiesta SSFP sequence was scanned along the short axis, covering apex to base of the left ventricle. In addition, the HeartVista real-time color phase-contrast GRE sequence was used to acquire the time-averaged flow in the ascending aorta at a cross section near the pulmonary vein (Figure 1). The Fiesta SSFP cardiac output (calculated using [stroke volume] X [heart rate]) was compared to the real-time PC-MRI cardiac output (calculated from aortic flow) for each subject. Measurements were compared using standard paired correlation and Bland-Altman analyses.

## Results

**Figure 2:**
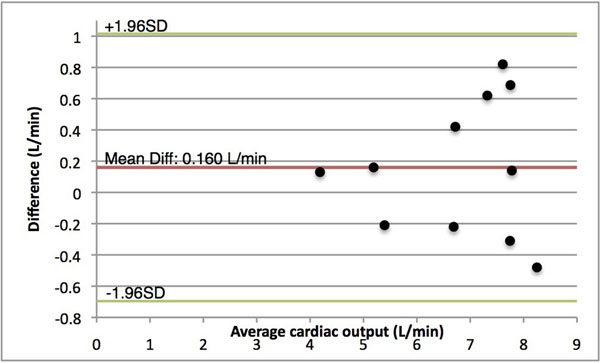
Bland-Altman plot comparing cardiac output computed using Fiesta SSFP and real-time PC-MRI.

The cardiac outputs of the real-time PC-MRI and Fiesta SSFP are well correlated with an overall R value of 0.8551, which improves to 0.966 with the elimination of one outlier. With the exception of this outlier, the range of error was between 4% to -8% using Bland-Altman analysis (Figure 2). The mean difference between cardiac outputs was 0.16 L/min (StdDev: 0.47 L/min). The Fiesta SSFP required 17-27 seconds to acquire each slice for a total acquisition time per study of 10-14 minutes. The real-time PC-MRI with resulting flow values was acquired and displayed at 21 frames per second. Accounting for region-of-interest placement, the real-time flow measurement and calculation was completed in less than 45 seconds.

## Conclusions

Utilizing a novel PC-MRI sequence that acquires and displays quantitative flow information in real-time, the accuracy of the real-time PC-MRI sequence has been validated with a small number of patients and volunteers. In addition, the real-time PC-MRI sequence required significantly less time to acquire images and calculate cardiac output than the conventional Fiesta SSFP.

## Funding

NIH 2R44HL084769.

